# Electrical detection of ppb region NO_2_ using Mg-porphyrin-modified graphene field-effect transistors†

**DOI:** 10.1039/d1na00519g

**Published:** 2021-07-29

**Authors:** Takashi Ikuta, Takashi Tamaki, Hiroshi Masai, Ryudai Nakanishi, Kitaro Endo, Jun Terao, Kenzo Maehashi

**Affiliations:** Division of Advanced Applied Physics, Institute of Engineering, Tokyo University of Agriculture and Technology 2-24-16, Nakacho Koganei Tokyo 184-8588 Japan ikuta@go.tuat.ac.jp; Department of Basic Science, Graduate School of Arts and Sciences, The University of Tokyo 3-8-1, Komaba Meguro-ku Tokyo 153-8902 Japan

## Abstract

The trace detection of NO_2_ through small sensors is essential for air quality measurement and the health field; however, small sensors based on electrical devices cannot detect NO_2_ with the desired selectivity and quantitativity in the parts per billion (ppb) concentration region. In this study, we fabricated metalloporphyrin-modified graphene field-effect transistors (FETs). Mg-, Ni-, Cu-, and Co-porphyrins were deposited on the graphene FETs, and the transfer characteristics were measured. With the introduction of NO_2_ in the ppb concentration region, the FETs of pristine graphene and Ni-, Cu-, and Co-porphyrin-modified graphene showed an insufficient response, whereas the Mg-porphyrin-modified graphene exhibited large voltage shifts in the transport characteristics. This indicates that Mg-porphyrin acts as an adsorption site for NO_2_ molecules. An analysis of the Dirac-point voltage shifts with the introduction of NO_2_ indicates that the shifts were well-fitted with the Langmuir adsorption isotherm model, and the limit of detection for NO_2_ was found to be 0.3 ppb in N_2_. The relationship between the mobility and the Dirac-point voltage shift with the NO_2_ concentration shows that the complex of NO_2_ and Mg-porphyrin behaves as a point-like charge impurity. Moreover, the Mg-porphyrin-modified graphene FETs show less response to other gases (O_2_, H_2_, acetic acid, trimethylamine, methanol, and hexane), thus indicating high sensitivity for NO_2_ detection. Furthermore, we successfully demonstrated the quantitative detection of NO_2_ in air, which is near the environmental standards. In conclusion, the results of the Mg-porphyrin-modified graphene FETs enable a rapid, easy, and selective detectability.

## Abstract 2

The Mg-porphyrin-modified graphene FETs showed NO_2_ detection at a low concentration of ppb order at room temperature and are highly selective toward NO_2_.

## Graphical abstract

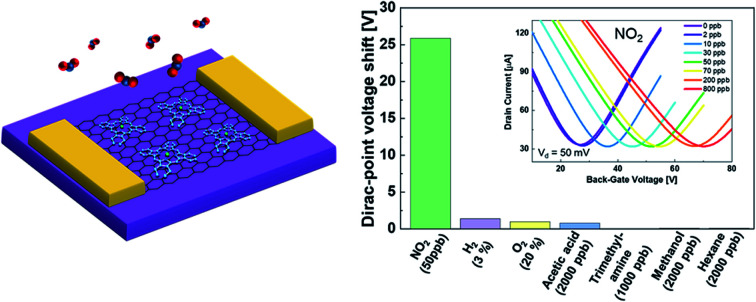

## Introduction

The selective detection of traces of harmful gases in an easy and rapid manner has become increasingly significant, especially in applications such as air quality measurement, security, and health care. In particular, inorganic acidic gases such as nitrogen dioxide (NO_2_) contribute the most to health and environmental problems.^1–4^ The quantitative detection of low concentrations of parts per billion (ppb) is essential to address these problems. For example, according to the air quality standards, the amount of NO_2_ cannot exceed 40–100 ppb in Japan, USA, and EU.^4–7^ However, such conventional trace and selective detections lack simplicity and quickness, because they require large and expensive facilities and preprocessing operations, such as separation and concentration. The use of gas chromatography and mass spectrometry for typical identification of trace harmful gases is one of the examples of a complex method.^8,9^ As a result, such conventional methods are not suitable for on-site and real-time measurements in applications in the abovementioned fields. Thus, the detection of NO_2_ at the ppb level in ambient air using small devices is necessary.

In recent decades, many research groups have studied and reported gas sensors based on metal-oxide semiconductors. The target gases mainly include organic gases (hydrocarbons and alcohols) and reductive inorganic gases (H_2_ and NH_3_) because the metal-oxide semiconductor uses the oxidation reactions at the gas–solid interface as the detection mechanism.^10,11^ However, metal-oxide semiconductors are not suitable for trace and selective detection of inorganic acidic gases as oxidants because they have low sensitivity and are easily disturbed by other gases. Moreover, metal-oxide semiconductor sensors can detect only in the sub-parts-per-million (ppm) region and require an external heating system to induce the oxidation reaction on their surface.^12–16^ Thus, developing methodologies for highly selective and sensitive gas sensors operating at room temperature for detecting trace amounts of inorganic acidic gases for practical applications is a major challenge.

In this study, we focused on a graphene device that was modified with porphyrin complexes to realize a sensor for inorganic acidic gases. Graphene is a two-dimensional material which is formed by carbon atoms, and it can be used in practical sensor devices that require high chemical stability and mobility.^17–20^ Owing to these benefits, several graphene-based sensors has been reported.^21–23^ However, pristine graphene sensors are not capable of quantitative and selective detection of target materials with a ppb order resolution. Some groups have reported that the introduction of defects into graphene could help overcome these challenges because the adsorption sites increase the sensitivity;^24–26^ however, in this method, various molecules are adsorbed on the defects in graphene and target molecules cannot be selected in principle. Furthermore, introducing defects into graphene degrades the electrical transport properties of graphene.^27,28^ Thus, the introduction of defects should be avoided when applying in electronic sensor devices. With regard to these factors, the modification of receptors on graphene has been extensively studied for improving the selectivity and sensitivity of graphene field-effect transistor (FET)-based gas sensors.^29–31^ Therefore, the limit of detection and selectivity of graphene-based sensors strongly depend on the receptor, and the best receptor must be selected for the detection of the target molecule. Among the available receptors, metalloporphyrins are preferred because they chemically recognize specific molecules as ligands, which can then be applied to chemical sensors with remarkable selectivity and sensitivity.^32–34^ In addition, the planar π-conjugated structure of metalloporphyrins helps modify the graphene surface by π–π interactions without inducing any defects in graphene. Therefore, the metalloporphyrin-modified graphene FET can detect the target gas with high selectivity and sensitivity through suitable combinations of porphyrins and the target gases.

In this study, we evaluated the effectiveness of metalloporphyrins as a receptor for inorganic acidic gases on a graphene device. We fabricated metalloporphyrin-modified graphene FETs for detecting NO_2_. Our experimental results show that large shifts were observed in the transfer characteristics of the graphene FETs with Mg-porphyrin with the introduction of NO_2_. The quantitative detection of NO_2_ at low concentrations in the ppb region was achieved using the Mg-porphyrin-modified graphene FETs in N_2_ or ambient air. Moreover, the use of Mg-porphyrin with a high affinity to oxygen atoms resulted in a remarkable selectivity toward NO_2_ as compared to other gases, including organic and reductive/oxidative gases.

## Experimental section

### Fabrication process of graphene field-effect transistors

**Fig. 1:**
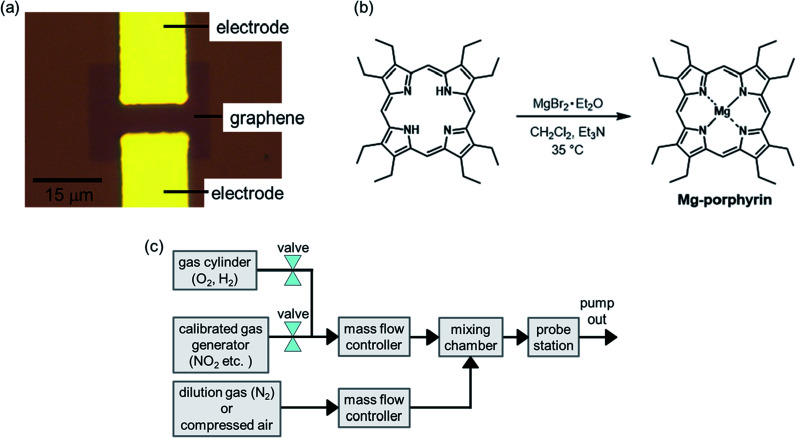
(a) Optical microscope image of the graphene FET, (b) reaction scheme of Mg-octaethylporphyrin, and (c) schematic image of the gas flow system.

First, Ti/Au electrodes were constructed on silicon substrates with 280 nm thermally oxidized SiO_2_ using photolithography and a lift-off process. Subsequently, monolayer graphene was grown on Cu foil by chemical vapour deposition, and it was transferred onto the substrate using the conventional transfer method.^35^ The excess graphene was etched with oxygen plasma to fabricate the graphene FETs. The channel length and width for the source and drain were approximately 5 and 15 μm, respectively (Fig. 1(a)). After the fabrication process, the graphene FETs were annealed at 300 °C in an Ar/H_2_ ambient atmosphere to remove resist residues on the graphene surface.

### Synthesis of metalloporphyrins

The Co, Cu, and Ni-octaethylporphyrins (Co, Cu, and Ni-porphyrins) were prepared according to previously reported procedures.^36,37^ The magnesium octaethylporphyrin (Mg-porphyrin) was synthesized using the following procedure. Octaethylporphyrin (74.5 mg, 0.140 mmol) was dissolved in CH_2_Cl_2_ (20 mL). Et_3_N (250 μL, 3.41 mmol) and magnesium bromide ethyl etherate (347 mg, 1.34 mmol) were added into the solution, and the mixture was stirred at 35 °C overnight before removing the solvent by evaporation (Fig. 1(b)). The residue was purified by column chromatography on basic alumina using CH_2_Cl_2_/Et_3_N (99.5 : 0.5 → 99 : 1) as the eluent. The resultant product was dissolved in MeOH, and water was added to the solution. The precipitate, which was collected and dried in a vacuum, yielded Mg-porphyrin as a purple solid (15.2 mg, 20% yield).

### Preparation of metalloporphyrin-modified graphene FETs and measurement setups

Metalloporphyrins (Mg, Ni, Cu, and Co) were dissolved in dichloromethane at a concentration of ∼10^−5^ M, and the graphene FETs were placed into the solution for 1 h to deposit metalloporphyrins on the graphene surface. After the deposition, the graphene FETs were dried using compressed air and placed in a vacuum chamber to carry out electrical measurements with a gas flow. Some target gases (NO_2_, acetic acid, methanol, hexane, and trimethylamine) were generated using a calibrated gas generator (Gastec, PD-1B) with N_2_ as the carrier gas. Other target gases (O_2_ and H_2_) were introduced from gas cylinders. These gases were diluted using two mass flow controllers (Horiba, SEC-E40) and a mixing chamber (Bronkhorst, gas mixing chamber), as shown in Fig. 1(c).^29^ In this study, the concentration of NO_2_ was adjusted to 2–800 ppb to evaluate response to NO_2_ near environmental standards. The transfer characteristics of the graphene FETs were measured at room temperature using a source measurement unit (Keysight Technologies, B2912).

## Results and discussion

### Characterization of metalloporphyrin-modified graphene FETs

**Fig. 2:**
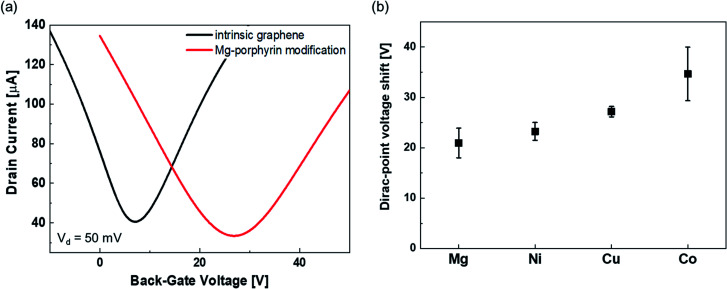
(a) Typical transfer characteristics in pristine graphene (black line) and Mg-porphyrin-modified graphene (red line). The transfer characteristics were measured by applying 50 mV with sweeping the back-gate voltage. (b) Amount of voltage shift at the Dirac point in the modification of Mg-, Ni-, Cu-, and Co-porphyrins. All error bars are the standard deviation of Dirac-point voltage shift for the four samples.

The typical transfer characteristics of the device with and without Mg-porphyrin are shown in Fig. 2(a). The transfer curve shifted in the positive direction after the Mg-porphyrin modification. A similar change was observed after the modification of other metalloporphyrins (Fig. 2(b)). Because the shift directions are the same, these shifts can be attributed to hole doping from the π-conjugated system of porphyrin to graphene.^38–40^ It is observed that the total voltage shift depends on the centre metal ions, which is presumably the difference in the electrostatic potential of the molecule. Based on these transfer characteristics, the hole and electron mobilities in the pristine graphene FET were calculated to be ∼4200 and 3200 cm^2^ V^−1^ s^−1^, respectively, and those of mobilities in the Mg-porphyrin-modified graphene FET are ∼2800 and 2400 cm^2^ V^−1^ s^−1^, respectively. The other metalloporphyrin-modified graphene FETs also show similar mobility. Normally, the mobility of graphene FETs decreases drastically when molecules are immobilized on graphene by a chemical reaction because the π-conjugated system in graphene changes to sp^3^ hybridization.^41,42^ Subsequently, the AFM images reveal that the surface morphology was slightly changed after the modification of the metalloporphyrins, and the surface roughness did not change significantly compared with the pristine graphene surface (Fig. S4†). The results indicate that based on the molecular structure, the metalloporphyrins were modified in parallel with the graphene surface to form a molecular membrane. Moreover, in this study, the high mobility was retained owing to physical adsorption by π–π interaction between a planar π-conjugated system in porphyrin and graphene without inducing defects (Fig. S5†).^40,43,44^ This is beneficial for FET-based sensors because high mobility leads to a clear signal. Therefore, a system consisting of graphene and π-conjugated materials is suitable for highly sensitive sensors.

### Response to NO_2_ in metalloporphyrin-modified graphene FETs in N_2_

**Fig. 3:**
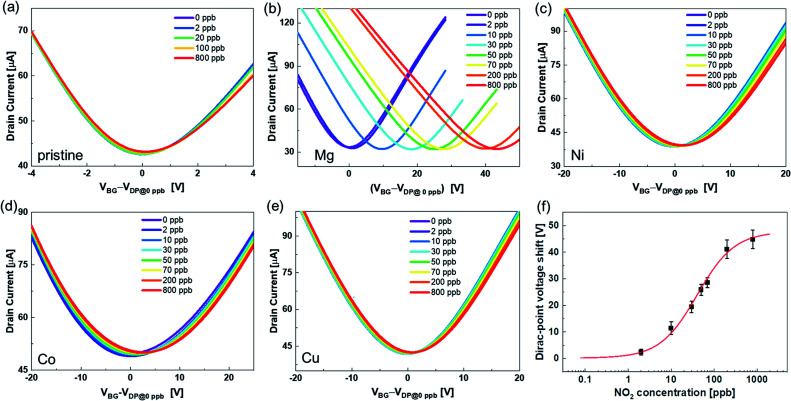
Transfer characteristics when NO_2_ was introduced into (a) pristine graphene and (b) Mg-, (c) Ni-, (d) Co-, and (e) Cu-porphyrin-modified graphene. These transfer characteristics were measured by applying the drain voltage of 50 mV with sweeping back-gate voltage. (f) NO_2_ concentration dependence of the Dirac-point voltage shift in the Mg-porphyrin-modified graphene FETs. Red line shows the curve fitted with the Langmuir adsorption isotherm. All error bars are the standard deviation of the Dirac-point voltage shift for the four samples.

Fig. 3 shows the transfer characteristics of the back-gated voltage (*V*_BG_–*V*_DP@0 ppb_) at NO_2_ concentrations from 0 to 800 ppb with N_2_ in the pristine graphene and metalloporphyrin-modified graphene FETs. Here, *V*_DP@0 ppb_ represents the Dirac-point voltage at a NO_2_ concentration of 0 ppb. In the graphene device, there was no clear shift in the transfer characteristics with the introduction of NO_2_ (Fig. 3(a)). Previous studies have shown that graphene with clean surfaces has no sensing capability, and first-principles calculations show a low adsorption energy between graphene and NO_2_.^45–48^ The results of this study are consistent with those of a previous study, and they imply that the graphene surface used in this study is clean and there are few adsorption sites on its surface. The pristine graphene FETs did not detect NO_2_ molecules. In contrast, in the Mg-porphyrin-modified graphene FET, the transfer characteristics shifted in the positive gate-voltage direction for all NO_2_ concentrations, as shown in Fig. 3(b). The sensor detected a low concentration of NO_2_ gas in the ppb region. In addition, the number of shifts increased with the NO_2_ concentration. However, the graphene FETs that were modified with other metalloporphyrins showed comparatively fewer shifts than those modified with Mg-porphyrin (Fig. 3(c)–(e)). The responsiveness of Mg-porphyrin to NO_2_ was possibly attributed to a coordination of NO_2_ with magnesium atoms as a Lewis acid or to a redox reaction with the porphyrin ring of Mg-porphyrin.^49–51^ Based on these results, it can be inferred that Mg-porphyrin is optimal for the receptor on the graphene FETs for detecting NO_2_. To evaluate the sensing capability for NO_2_ detection, the Dirac-point voltage (*V*_DP_) shift as a function of NO_2_ concentration is shown in Fig. 3(f). The data were then analysed using the Langmuir adsorption isotherm [eqn (1)].^35,44^1
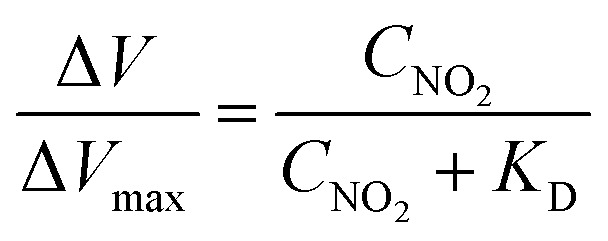
where Δ*V*, Δ*V*_max_, *C*_NO_2__, and *K*_D_ are the voltage shift, maximum voltage shift, concentration of NO_2_, and dissociation constant, respectively. The solid line in Fig. 3(d) shows a fitting curve with the Langmuir isotherm. As shown in Fig. 3(d), the measurement data were well fitted to the Langmuir adsorption isotherm. This suggests that one porphyrin molecule captures one NO_2_ molecule in the device system with the basic assumptions of the Langmuir adsorption model. From this result, the *K*_D_ and limit of detection (signal/noise = 3) were found to be 41 and 0.4 ppb, respectively, as shown in Fig. S6.† Here, noise was defined as the standard deviation of the measurement at 0 ppb, and it was found to be 134 mV. Based on the aforementioned factors, it can be inferred that the Mg-porphyrin-modified graphene FETs derive high quantification from the Langmuir adsorption isotherm in the trace concentration regions from sub-ppb to ppm order.

### Evaluation of the sensing mechanism in the Mg-porphyrin-modified graphene FETs from the relationship between mobility and the Dirac-point voltage shift

**Fig. 4:**
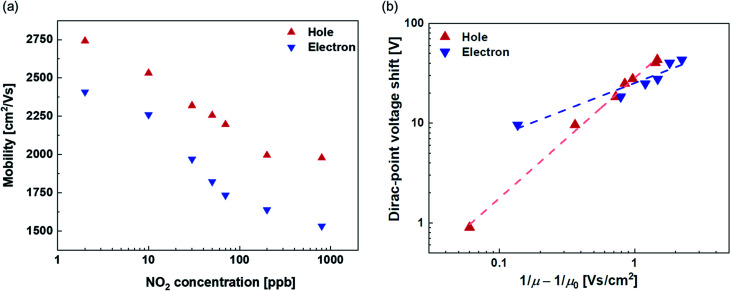
(a) NO_2_ dependence of the mobility for holes (red) and electrons (blue) in the Mg-porphyrin-modified graphene FET. (b) The Dirac-point voltage shift *vs.* 1/*μ* − 1/*μ*_0_ in the Mg-porphyrin-modified graphene FET. Dashed lines represent power-law fits to the equation (the Dirac-point voltage shift) ∼ (1/*μ* − 1/*μ*_0_)^*b*^, where *b* is 1.2 and 0.5 for holes (red) and electrons (blue), respectively. Evaluation of selectivity in the Mg-porphyrin-modified graphene FETs.

Fig. 4(a) shows the NO_2_ concentration dependence of the field-effect mobility. Both hole and electron mobilities decrease with the increase in the NO_2_ concentration. It is assumed that the complexes of Mg-porphyrin and NO_2_ molecules caused the scattering increase by NO_2_ adsorption in this system.^52^ To investigate the scattering effect, the Dirac-point voltage shift is plotted *vs.* (1/*μ* − 1/*μ*_0_) in Fig. 4(b).^53^ Here, *μ* and *μ*_0_ represent the mobility at each NO_2_ concentration and that at 0 ppb, respectively. The dashed lines represent fitting lines that are derived from the power law relationship of the Dirac-point voltage shift ∼ (1/*μ* − 1/*μ*_0_)^*b*^, with values of *b* = 1.2 and 0.5 for holes and electrons, respectively. The decrease in the hole mobility with the introduction of NO_2_ is similar to the case with point-like charge impurities (1/*r* Coulomb scattering) from the adsorbed metal atoms on graphene.^54–57^ Therefore, the Mg-porphyrin captures NO_2_ molecules, and the complexes of NO_2_ and Mg-porphyrin act as point-like charge impurities. The change in the electron mobility exhibits a gentler slope than that of the hole mobility with respect to the change in the Dirac-point voltage shift. This indicates that the Mg-porphyrin and NO_2_ complexes are less likely to be scattered for electrons. These factors caused a shift in the transfer characteristics of the Mg-porphyrin-modified graphene FETs. These results indicate that the complexes act as charge impurities and enable the generation of electrical signals in the Mg-porphyrin-modified graphene FETs by the adsorption of NO_2_. Thus, the modification of Mg-porphyrin on the graphene surface in the graphene FETs helped detect NO_2_ with a large voltage shift.

### Evaluation of selectivity in the Mg-porphyrin-modified graphene FETs

**Fig. 5:**
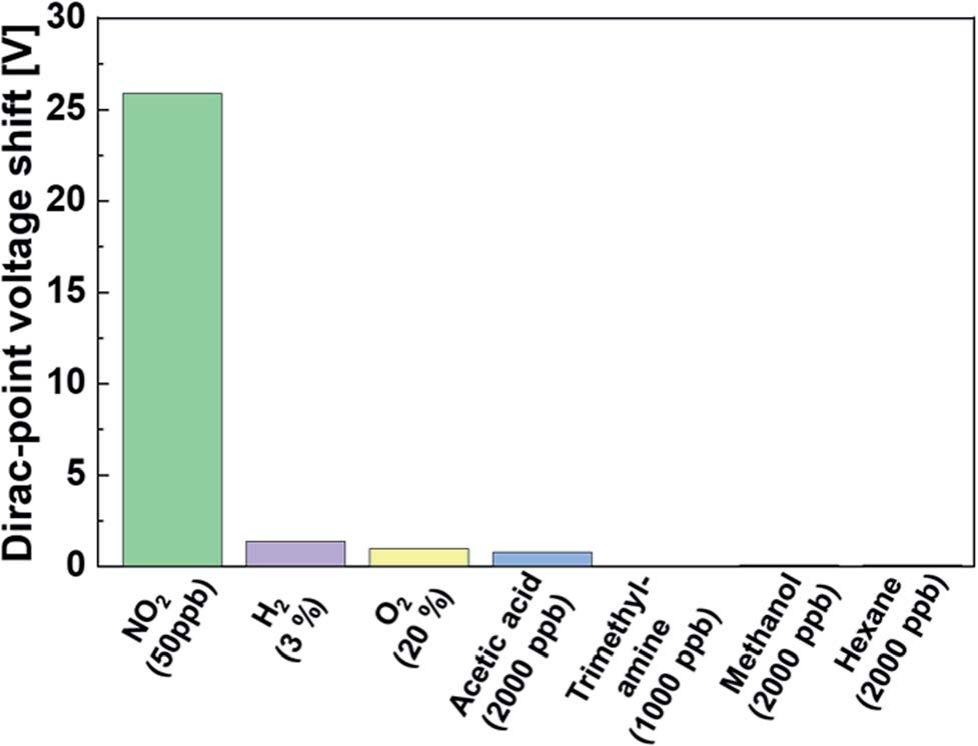
Shifts in the Dirac-point voltage due to different gases at each concentration in the Mg-porphyrin-modified graphene FETs. (NO_2_: 50 ppb, H_2_: 3%, O_2_: 20%, acetic acid: 2000 ppb, trimethylamine: 1000 ppb, methanol: 2000 ppb and hexane: 2000 ppb).

To investigate the selectivity of the NO_2_ gas sensor, we performed the same measurements with different gases. The voltage shift of the Dirac point in the graphene-based sensor was characterized by different target gases for all concentrations at room temperature. As shown in Fig. 5, the Dirac-point voltage shift in NO_2_ detection was over ten times higher than that of other gases, and the concentration of NO_2_ was lower than that of other gases by over two orders of magnitude. The sensor did not respond to O_2_ and H_2_ gases, and the unresponsiveness to oxidative and reductive gases on the graphene-based sensor was a specific feature as compared to typical sensors that utilized redox-based metal-oxide semiconductors. In addition, under typical volatile organic gases such as hydrocarbon (hexane) and alcohol (methanol), no voltage shift was observed in the Dirac point. It is worth noting that the sensor exhibited high selectivity to inorganic acidic gas (NO_2_) rather than organic acidic gas (acetic acid) and an organic basic gas (trimethylamine). The results indicate that the Mg-porphyrin-modified graphene FET can potentially be used for the detection of NO_2_ with high selectivity as compared to other gas sensors with conventional detection mechanisms. This difference in detectability is due to the different magnitudes of interaction between the Mg porphyrin and each target molecule.

### Quantitative detection of NO_2_ using Mg-porphyrin-modified graphene FETs in ambient air

**Fig. 6:**
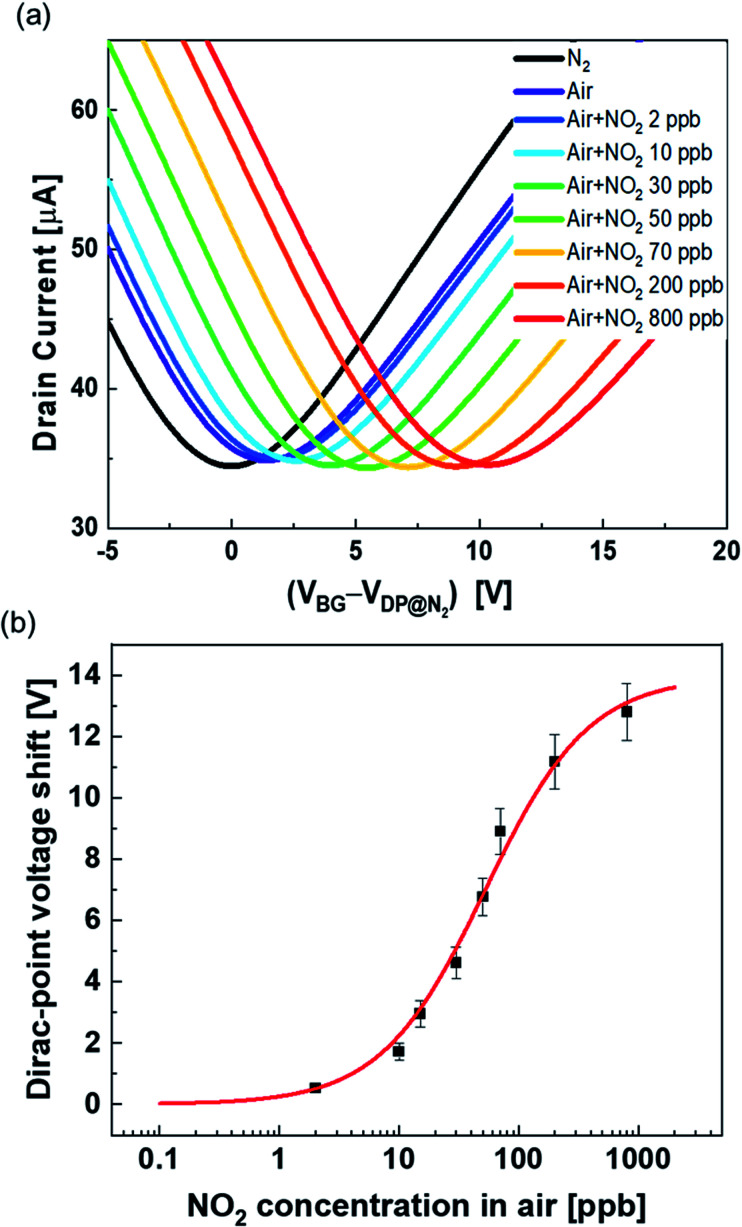
(a) Transfer characteristics when NO_2_ with air was introduced into the Mg-porphyrin-modified graphene FET. The transfer characteristics were measured by applying 50 mV with sweeping the back-gate voltage. (b) NO_2_ concentration dependence of the Dirac-point voltage shift in air in the Mg-porphyrin-modified graphene FETs. Red line shows the curve fitted with the Langmuir adsorption isotherm. All error bars are the standard deviation of the Dirac-point voltage shift for the three samples.

Fig. 6(a) shows the transport characteristics of the Mg-porphyrin-modified graphene FET with the introduction of NO_2_ with compressed air. The shift in the positive axis was observed with the introduction of air, which is attributable to the adsorption of oxygen, carbon dioxide or water molecules in the air. The shift was caused by the adsorption of these molecules in air saturated within one hour (Fig. S7†). Despite the atmospheric conditions, a distinct shift was observed in the transfer characteristics with the introduction of NO_2_ in air, similar to the case of NO_2_ in N_2_. The same analysis as in the above discussion of the Langmuir adsorption isotherm shows that the dissociation constant, Δ*V*_max_, and limit of detection in air were calculated to be 52 ppb, 14 V, and 1.4 ppb, respectively (Fig. 6(b)). Δ*V*_max_ in air was lower than that in N_2_. This is mainly due to the adsorption of molecules in the atmosphere. Furthermore, the effect of NO_2_ adsorption is reduced by screening.^58,59^ However, the values in air show that the Mg-porphyrin-modified graphene FETs are capable of quantifying the concentration of NO_2_ near the environmental standards of NO_2_ even under atmospheric conditions,^5–7^ thus indicating that the Mg-porphyrin-modified graphene FETs are useful in air quality measurement.

## Conclusions

We fabricated Mg-porphyrin-modified graphene FETs for NO_2_ detection. The Mg-porphyrin-modified graphene FETs showed NO_2_ detection at a low concentration of ppb order at room temperature. Pristine graphene is unaffected upon exposure to NO_2_. The modification of Mg-porphyrin onto graphene helps detect NO_2_ because NO_2_molecules are captured and transduced to electrical signals. The Dirac-point shift with NO_2_ concentration is well-fitted with the Langmuir isotherm, and then the Mg-modified graphene FETs quantitatively detect NO_2_ based on the Langmuir model. The Mg-porphyrin-modified graphene FETs are highly selective toward NO_2_. Moreover, we successfully demonstrated the quantitative detection of NO_2_ at low concentrations in the ppb region in air using the Mg-porphyrin-modified graphene FETs. Our results indicate that the Mg-porphyrin-modified graphene FETs are robust for the detection of NO_2_ and are useful for air quality measurement with electronic devices.

## Author contributions

Takashi Ikuta: conceptualization, data curation, formal analysis, validation, investigation, writing – original draft, and funding acquisition. Takashi Tamaki: data curation, formal analysis, investigation, validation, writing – review & editing, and funding acquisition. Hiroshi Masai: data curation, formal analysis, investigation, validation, writing-review & editing, and funding acquisition. Ryudai Nakanishi: investigation. Kitaro Endo: investigation. Jun Terao: writing – review & editing, funding acquisition, and supervision. Kenzo Maehashi: writing – review & editing, funding acquisition, and supervision.

## Conflicts of interest

There are no conflicts to declare.

## Supplementary Material

NA-003-D1NA00519G-s001
