# Supplementary material for: Electrical detection of ppb region NO2 using Mg-porphyrin-modified graphene field-effect transistors
Source: Nanoscale Adv. 2021 Jul 29;3(20):5793–800. doi: 10.1039/d1na00519g (PMC9417097; doi:10.1039/d1na00519g)
Supplement: NA-003-D1NA00519G-s001 [file NA-003-D1NA00519G-s001.pdf]

Electronic Supplementary Material (ESI) for Nanoscale Advances.
This journal is © The Royal Society of Chemistry 2021

Supporting Information

Electrical Detection of ppb Region NO_2 using Mg-porphyrin-modified Graphene Field-effect Transistors

Takashi Ikuta, Takashi Tamaki, Hiroshi Masai, Ryudai Nakanishi, Kitaro Endo, Jun Terao, Kenzo Maehashi

Table of Contents

Experimental Details

Figure S1: ^{1}H NMR spectrum (500 MHz, $CDCl_3$, r.t.) of Mg-porphyrin

Figure S2: ^{13}C NMR spectrum (126 MHz, $CDCl_3$, r.t.) of Mg-porphyrin.

Figure S3: (top) Experimental and (bottom) calculated ESI-MS spectrum of Mg-porphyrin

Figure S4: AFM images before and after metalloporphyrin-modification on graphene.

Figure S5: Raman spectra of pristine and metalloporphyrin-modified graphene.

Figure S6: Langmuir plots of Dirac point shift with NO_2 concentration in the range from 0.1 to 5 ppb.

Figure S7: (left) Transfer characteristics of the Mg-porphyrin-modified graphene FET when air was induced. (right) Time dependence of Dirac point voltage shift in ambient air.

Figure S8: Transfer characteristics before and after NO_2 detection.

Experimental Details

General remarks

Unless otherwise stated, commercially available chemicals were used as received. CH_2Cl_2 was purchased from Kanto Chemical and further purified by passage through activated alumina under positive argon or nitrogen pressure.[1] Electrospray ionization (ESI) mass spectra were obtained on a BRUKER micrOTOFII-KEO2 System. ^{1}H NMR (500 MHz) and ^{13}C $\{^{1}H\}$NMR (126 MHz) were measured with a Bruker AVANCE-500 spectrometer. The ^{1}H NMR chemical shifts are reported relative to residual protonated solvents (7.26 ppm) in $CDCl_3$. The ^{13}C NMR chemical shifts are reported relative to $^{13}CDCl_3$ (77.16 ppm).

Spectrum data of Mg-porphyrin

ESI-TOF MS (*m/z*): [Mg-porphyrin]$^+$ Calcd for $C_{36}H_{44}MgN_4$ 556.3; Found 556.2

^{1}H NMR (500 MHz, $CDCl_3$, r.t.): δ_H= 10.12 (s, 4H, *meso*-H), 4.13 (q, *J* = 7.7 Hz, 16H, CH_2), 1.95 (t, *J* = 7.7 Hz, 24H, CH_3)

^{13}C NMR (126 MHz, $CDCl_3$, r.t.): δ_C= 147.32, 142.25, 97.89, 20.12, 18.97

Reference

1) Pangborn, A. B.; Giardello, M. A.; Grubbs, R. H.; Rosen, R. K.; Timmers, F. J. *Organometallics* **1996**, *15*, 1518-1520.

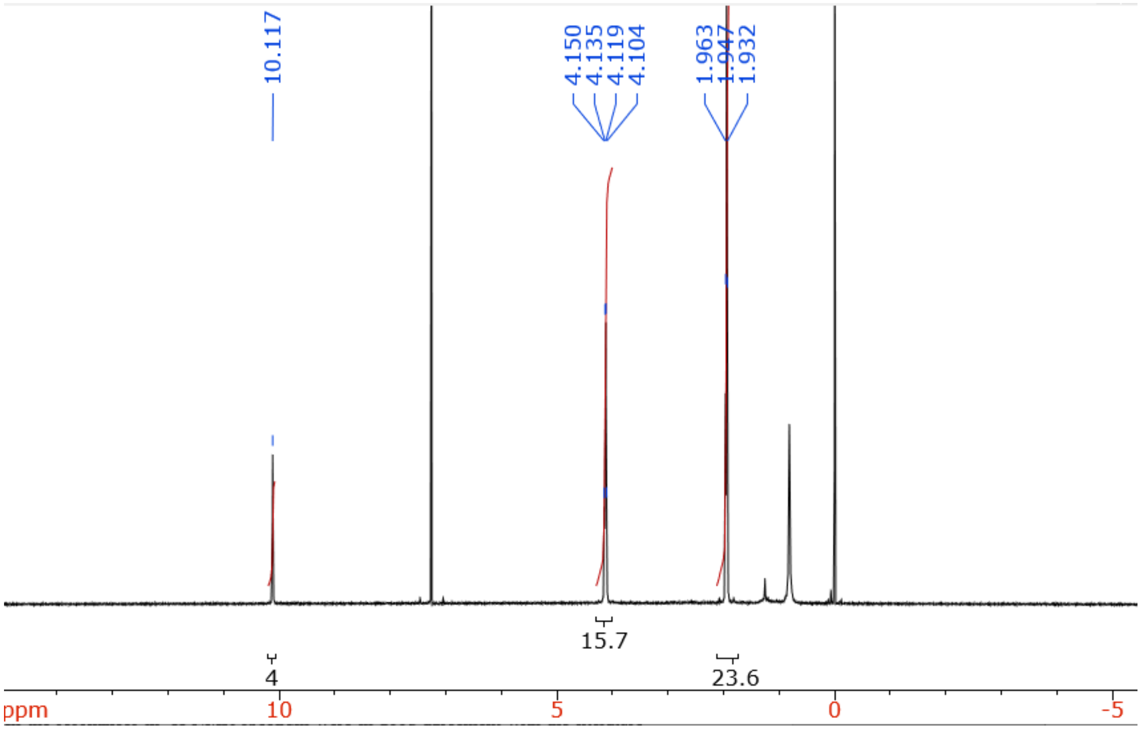

Figure S1: ^{1}H NMR spectrum (500 MHz, $CDCl_3$, r.t.) of Mg-porphyrin.

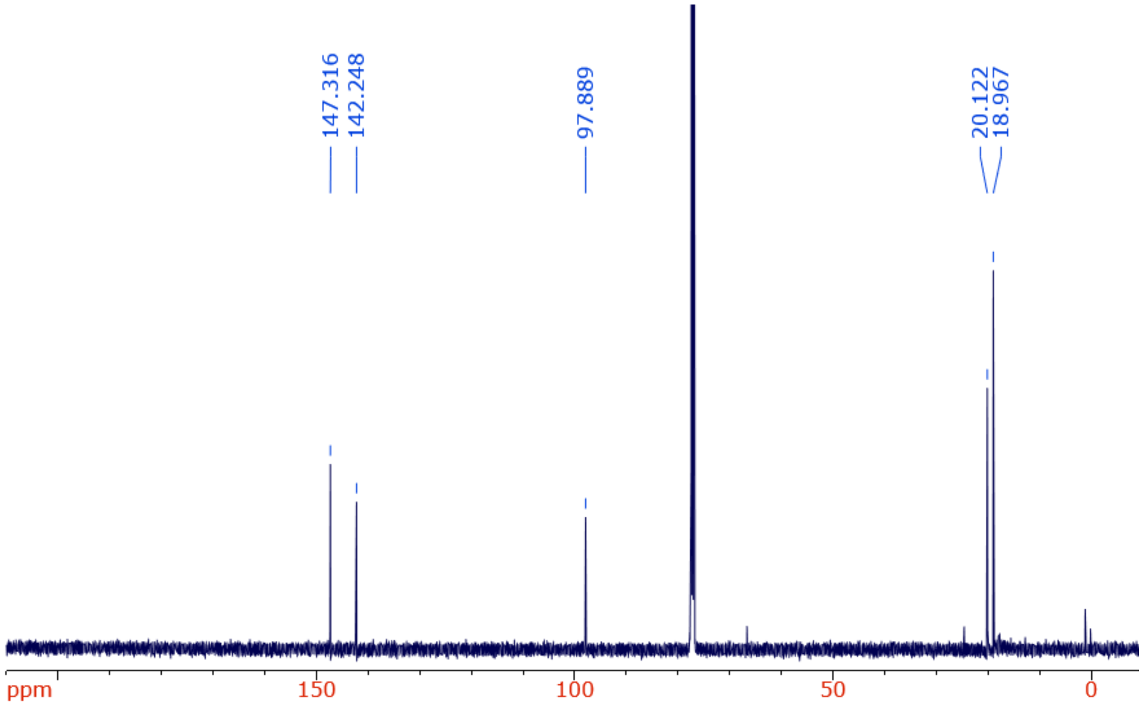

Figure S2: ^{13}C NMR spectrum (126 MHz, $CDCl_3$, r.t.) of Mg-porphyrin.

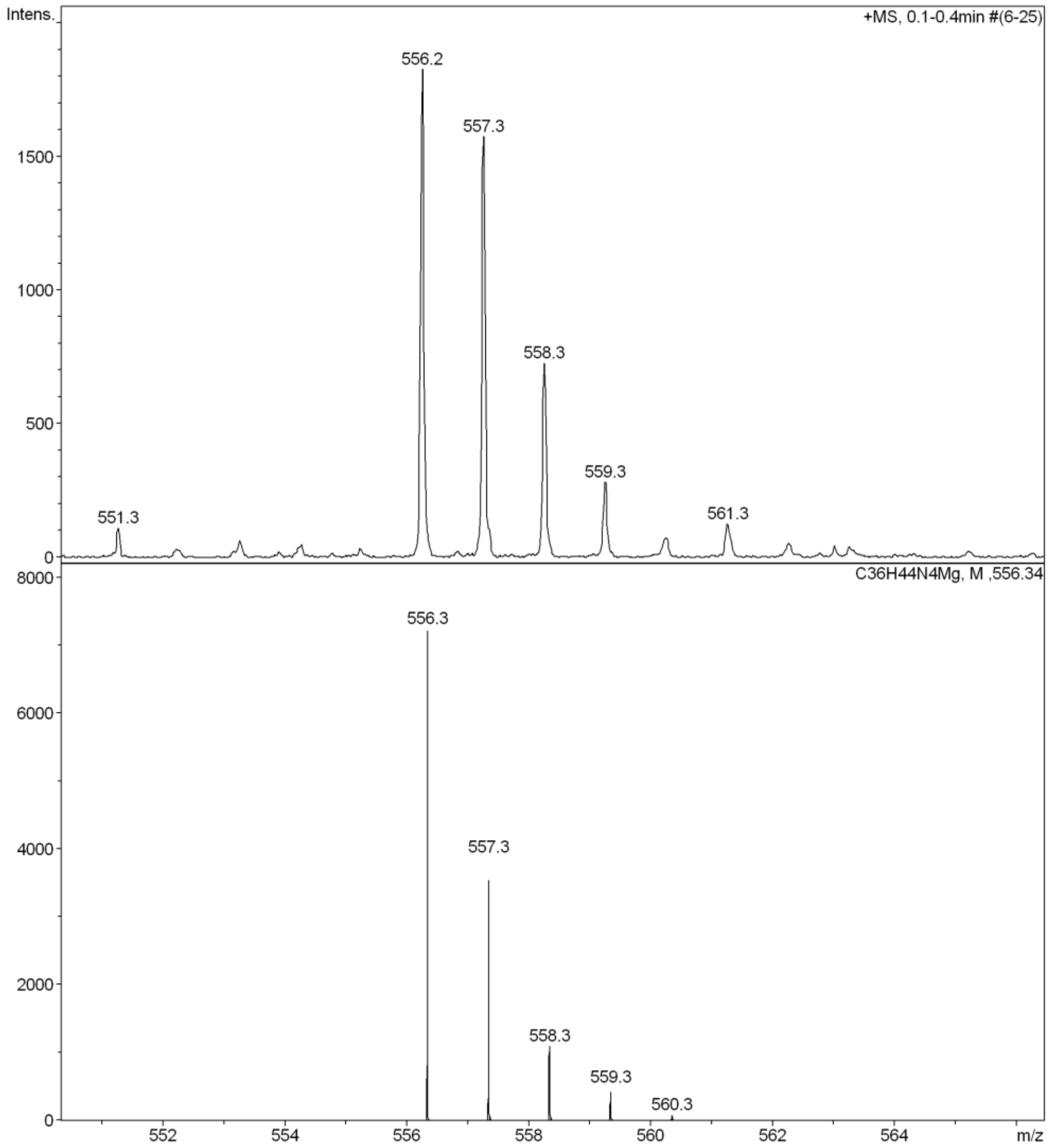

Figure S3: (top) Experimental and (bottom) calculated ESI-MS spectrum of Mg-porphyrin.

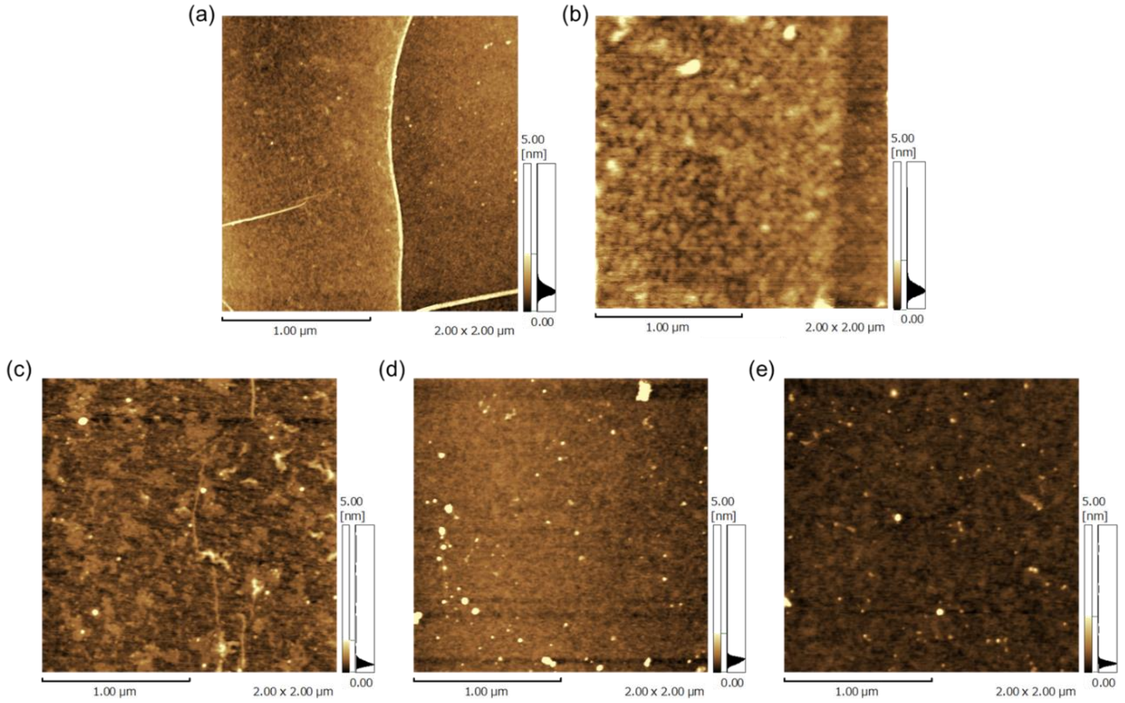

Figure S4: AFM images of (a) pristine, (b) Mg-porphyrin-modified, (c) Co-porphyrin-modified, (d) Ni-porphyrin-modified, and (e) Cu-porphyrin-modified graphene. The root mean square roughness in each case is 0.250, 0.378, 0.637, 0.513, and 0.352, respectively.

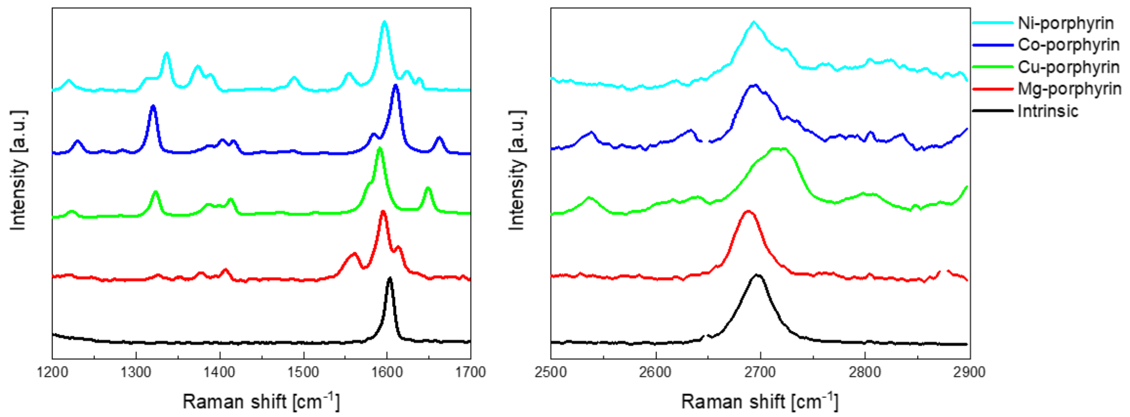

Figure S5: Raman spectra of pristine and metalloporphyrin-modified graphene. (left) 1200~1700 cm^{-1} and (right) 2500~2900 cm^{-1}. Each spectrum is normalized by a maximum value.

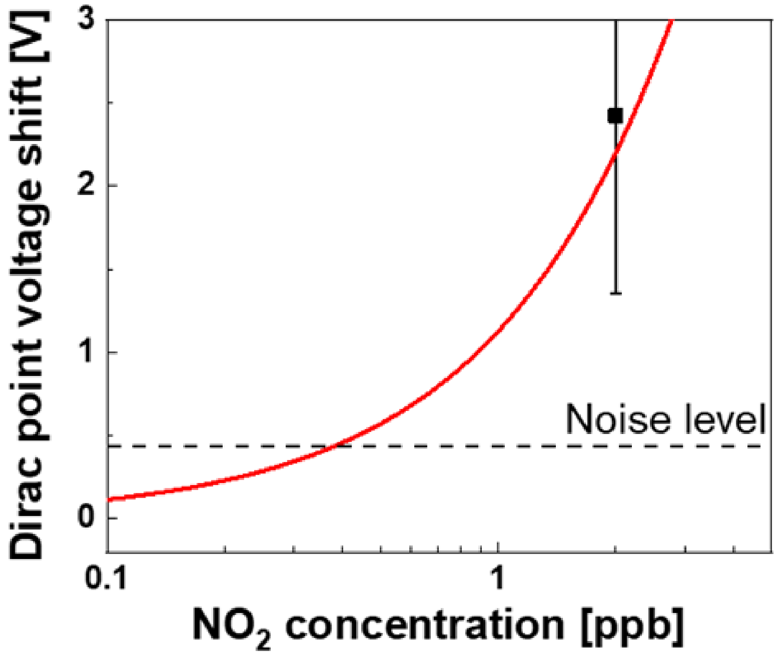

Figure S6: NO_2 concentration dependence of the Dirac-point voltage shift in the Mg-porphyrin-modified graphene FETs. The noise level (S/N = 3) was calculated to be ~430 mV from standard deviation of measurement at 0 ppb.

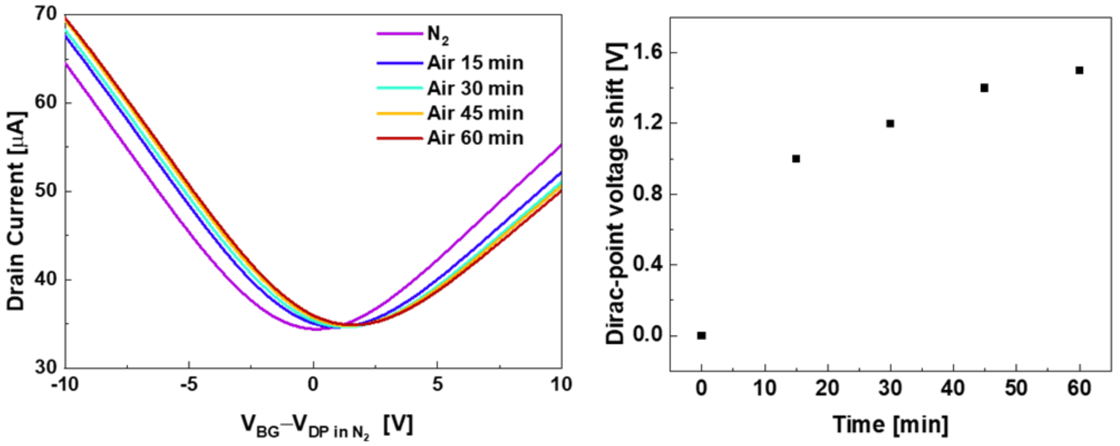

Figure S7: (left) Transfer characteristics of the Mg-porphyrin-modified graphene FET when air was induced. The transfer characteristics were measured by applying drain voltage of 50 mV with sweeping back-gate voltage. (right) Time dependence of the Dirac point voltage shift in ambient air.

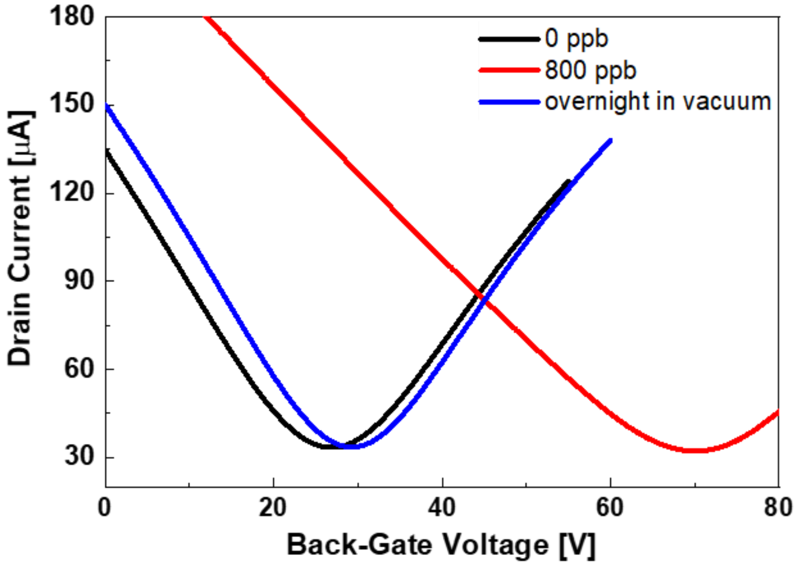

Figure S8: Transfer characteristics of the Mg-porphyrin-modified graphene FET when induced NO_2 concentration are (black) 0 ppb, (red) NO_2 800 ppb, and (blue) 0 ppb after overnight in vacuum.
